# Efficacy and Safety of Intranasal Esketamine in Treatment-Resistant Depression in Adults: A Systematic Review

**DOI:** 10.7759/cureus.17352

**Published:** 2021-08-21

**Authors:** Alisha Sapkota, Hajra Khurshid, Israa A Qureshi, Nasrin Jahan, Terry R Went, Waleed Sultan, Michael Alfonso

**Affiliations:** 1 Psychiatry, California Institute of Behavioral Neurosciences & Psychology, Fairfield, USA; 2 Internal Medicine, California Institute of Behavioral Neurosciences & Psychology, Fairfield, USA

**Keywords:** adverse event, esketamine, ketamine, treatment-resistant depression, intranasal

## Abstract

Intranasal form of esketamine, the S-enantiomer of racemic ketamine, was approved by the US FDA in 2019 for treatment-resistant depression (TRD) in adults. Since intranasal esketamine is a newly approved drug with a novel mechanism of action, much still remains unknown in regard to its use in TRD. The objective of this study is to systematically review the latest existing evidence on intranasal esketamine, and provide a better insight into its safety and efficacy in TRD in adults.

PubMed, MEDLINE (through PubMed), and Google Scholar were systematically searched from 2016 to 2021, using automation tools. After removal of duplicates and screening on the basis of title/abstract, eligibility criteria were applied and quality appraisal was done independently by two reviewers.

A total of 10 studies were selected for the final review which included five clinical trials (three short-term trials, one withdrawal design relapse prevention study, and one long-term study), three post hoc studies, one case/non-case study, and one review article. Out of three short-term clinical trials, only one demonstrated a statistically significant difference between treatment with esketamine plus oral antidepressant (OAD) vs placebo plus OAD. The result of the relapse prevention study showed significantly delayed relapse of depressive symptoms in esketamine plus OAD arm when compared to placebo plus OAD arm. Similarly, the result of the long-term clinical trial showed that the improvement in depressive symptoms was found to be sustained in those using esketamine. The most common adverse effects of esketamine included nausea, dizziness, dissociation, headache, vertigo, somnolence, and dysgeusia (altered sense of taste); most were mild-moderate in severity. One case/non-case study reported rare adverse effects including panic attacks, mania, ataxia, akathisia, self-harm ideation, increased loquacity (talkativeness), and autoscopy.

Intranasal esketamine has shown efficacy in reducing depressive symptoms in clinical trials, but the clinical meaningfulness of the treatment effect in the real-world population still needs to be explored. Although the safety profile of esketamine appears to be favorable in most clinical trials, some serious side effects are being reported to the FDA Adverse Event Reporting System, and therefore requires further investigation. More robust clinical trials, especially long-term randomized controlled trials are needed which can help provide a better assessment on the efficacy and safety of intranasal esketamine in the treatment of TRD.

## Introduction and background

Major depressive disorder (MDD) is a common psychiatric condition affecting around 264 million people worldwide [1]. In the United States alone, an estimated 7.1% of the adult population (equivalent to 17.3 million adults) were reported to have at least one major depressive episode in 2017 [2]. MDD can impair psychosocial functioning and is one of the common antecedents of suicide [1,3]. Biogenic amine antidepressants are effective medications for treating MDD, albeit with limitations; one of which being the delayed onset of effect ranging from four to six weeks. During this time, patients can remain symptomatic and are at risk of developing suicidal tendencies, which pose a major challenge to the treatment [4-5]. Furthermore, around one-third of patients with MDD do not respond to antidepressant therapy and eventually may develop treatment-resistant depression (TRD) [6].

Despite a lack of consensus definition, TRD has been commonly defined as the failure of patients to respond to at least two different antidepressants given at an adequate dose and duration, in the current depressive episode [7-8]. The management of TRD can be complex and difficult. It involves the use of multiple strategies such as switching therapies to a different antidepressant class; augmentation therapy using lithium, second-generation antipsychotics, and triiodothyronine; electroconvulsive therapy (ECT); and psychotherapeutic approach. More recently, ketamine, psilocybin, and anti-inflammatories are being considered as novel therapeutics [9-10].

Esketamine is the S-enantiomer of racemic ketamine and is found to have three to four times more affinity for N-methyl-D-aspartate (NMDA) receptors than R-enantiomer of ketamine (arketamine), thus making esketamine efficient even at a lower dose [11]. Intranasal form of esketamine was approved by the FDA for the treatment of TRD in adults in 2019 [12]. Since there’s always been a growing need for new effective treatments for TRD, the approval of intranasal esketamine has received quite a momentum. While many have praised and welcomed esketamine as a novel therapy for TRD, other experts have raised questions regarding the legitimacy of its efficacy and safety in the real-world population [13-15]. In the light of these new concerns, our research aims to assess further and add to the existing knowledge about esketamine, regarding its efficacy and safety, by conducting a systematic review using the latest existing evidence.

## Review

Materials and Methods

Search Strategy

The databases such as PubMed, MEDLINE (through PubMed), and Google Scholar were systematically searched for collecting data. We explored the PubMed database with the help of Medical Subject Heading (MeSH) terms and keywords: Esketamine, Intranasal Esketamine, Treatment Resistant Depression. We performed an automated search (with the application of filters) on April 16, 2021, and came across 498 articles in PubMed. The details regarding the search strategies are described in Table 1.

**Table 1:** Summary of the entire search strategy used for the review MeSH: Medical subject heading

Search strategy	Database	Total number of articles	Total number of articles with automation tools	After removal of duplicates and screening	After full screening and quality appraisal
Advanced search: Esketamine OR Intranasal Esketamine OR “Esketamine” [Supplementary Concept] AND Treatment Resistant Depression OR (“Depressive Disorder, Treatment-Resistant/drug therapy”[MesH] OR “Depressive Disorder, Treatment-Resistant/therapy”[MesH])	PubMed	1395	498	14	7
Keyword: Esketamine and Treatment Resistant Depression	Google Scholar	1990	1710	29	3

Inclusion and Exclusion Criteria

Inclusion criteria: (a) study type: phase-three clinical trials, post hoc studies, observational studies and reviews (systematic reviews and narrative reviews); (b) language: English; (c) patients who are 18 years and above, with TRD; (d) intervention: intranasal esketamine (given along with oral antidepressant (OAD)).

Exclusion criteria: animal studies, phase one and two clinical trials, gray literature, articles in languages other than English, case reports and series, letters to the editor, and studies published before 2016.

Results

We obtained a total of 2208 articles after searching through databases using automation tools. Records were then screened on the basis of title and abstract, duplicates were removed, and 43 articles were retrieved. After applying inclusion/exclusion criteria and quality appraisal, we had a total of 10 articles. Two reviewers (AS and HK) went through screening process, quality assessment, and data extraction independently. Quality assessments were performed using the following tools:

Randomized controlled trials (RCT)= Cochrane Risk of Bias tool

Quasi-experimental study= Joanna Briggs Institute (JBI) critical appraisal checklist for quasi-experimental study

Observational studies= The Newcastle-Ottawa Scale (NOS)

Systematic review and meta-analysis= A MeaSurement Tool to Assess systematic Reviews (AMSTAR)

Narrative review= Scale for the Assessment of Narrative Review Articles (SANRA)

**Figure 1:**
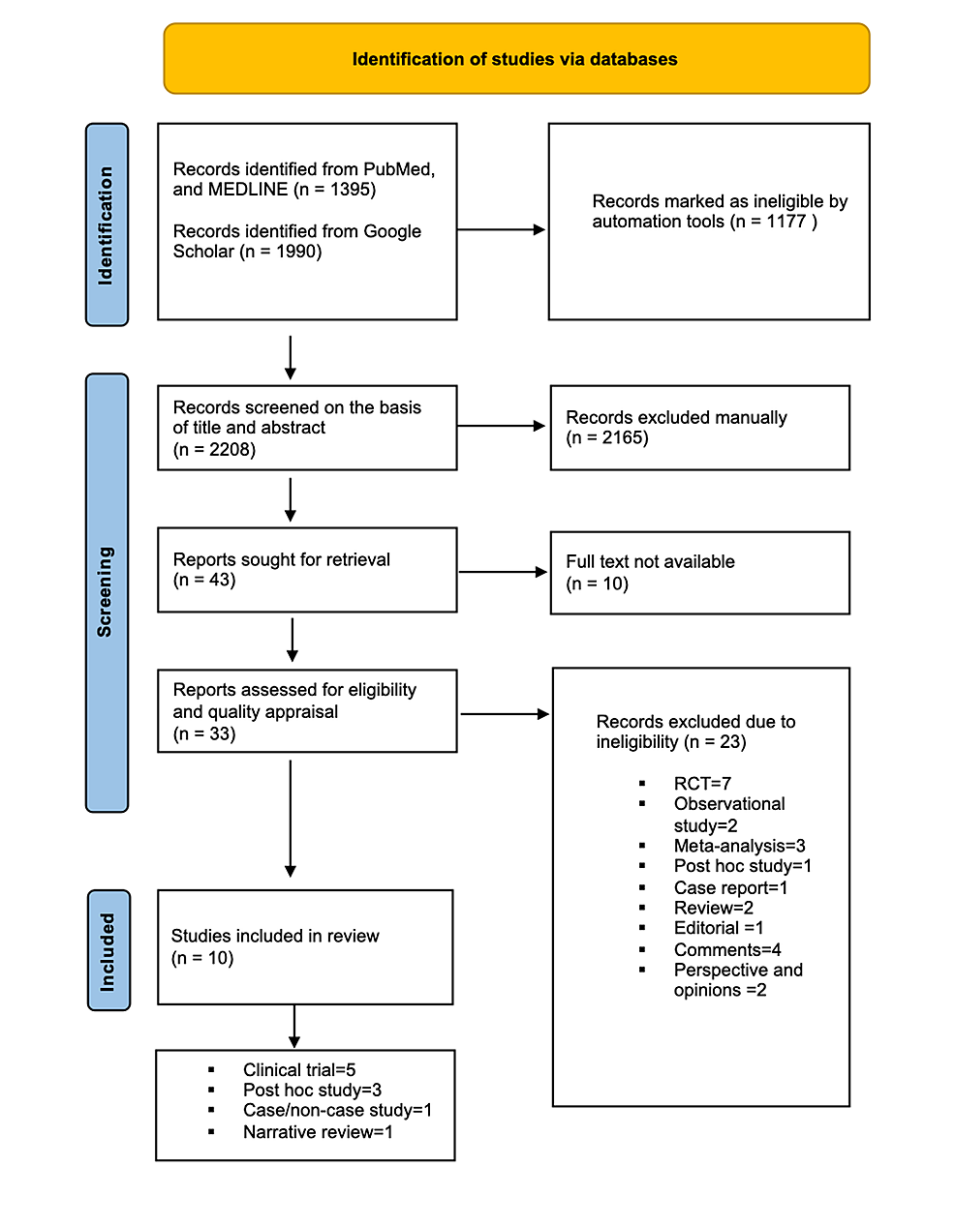
PRISMA flow diagram 2020 PRISMA: Preferred reporting items for systematic review and meta-analysis

Figure 1 shows the PRISMA flow diagram [16] which demonstrates the steps taken during the conduction of the search and the final articles included.

Tables 2, 3 summarize the characteristics of the studies included. We included a total of 10 studies which consisted of five phase-three clinical trials, three post-hoc studies, one case/non-case study, and one narrative review.

**Table 2:** Summary of phase-three clinical trials of esketamine ESK: esketamine; OAD: oral antidepressant; MADRS: Montgomery-Asberg Depression Rating Scale; CSSRS: Columbia-Suicide Severity Rating Scale; CADSS: Clinician-Administered Dissociative States Scale; BPRS+: Brief Psychiatric Rating Scale (4-item Positive Symptom Scale); MOAA/S: Modified Observer’s Assessment of Alertness/Sedation Scale; PWC-20: Physician Withdrawal Checklist; TRD: treatment-resistant depression; BPIC-SS: Bladder Pain/Interstitial Cystitis Symptom Score; TEAEs: treatment-emergent adverse events *MADRS total score ranges from 0 to 60. A higher score indicates increased severity of depression, whereas a negative change in score is indicative of improvement. **TRD: Nonresponse (≤ 25% improvement in MADRS) to at least two OAD given at an adequate dose in the current depressive episode for at least six weeks duration. ***Stable remission: MADRS total score ≤12 for at least three weeks in the last four weeks. ****Stable response without remission: MADRS score reduction by ≥50% from the baseline seen in the last two weeks of optimization phase but without acquiring remission.

Author/Year of publication/Name of study	Study details	Study population and duration	Dosing	Results	
				Efficacy	Safety
Fedgchin et al. (2019) [17] TRANSFORM-1	Randomized, multi-center, double-blind, and active-controlled; fixed dosing; arms = ESK (56 mg or 84 mg) plus OAD vs placebo plus OAD; N = 346 (56 mg ESK plus OAD = 117; 84mg ESK plus OAD = 116; placebo plus OAD = 113)	Adults with TRD**; age group = 18 to 64; treatment phase of four weeks, follow up for 24 weeks or entry into SUSTAIN-1	ESK 56 mg or 84 mg given intranasally two times per week	MADRS was used to assess efficacy, primary efficacy endpoint being a change in MADRS total score* from baseline (day one) to day 28. No statistically significant difference was seen between treatment with ESK plus OAD group compared to placebo plus OAD group	Safety assessment performed via physical examination, nasal examination, cognitive testing, CSSRS, CADSS, BPRS+, MOAA/S, PWC-20, Global Assessment of Discharge Readiness. Most common side effects included nausea, dizziness, dissociation, headache, and vertigo
Popova et al. (2019) [18] TRANSFORM-2	Randomized, multi-center, double-blind, and active-controlled; flexible dosing; arms = ESK (56 mg or 84 mg) plus OAD vs placebo plus OAD; N = 223 (ESK plus OAD = 114; placebo plus OAD = 109)	Adults with TRD; age group = 18 to 64; treatment phase of four weeks, follow up for 24 weeks or entry into SUSTAIN-1	ESK 56 mg or 84 mg given intranasally two times per week	MADRS used to assess efficacy, primary efficacy endpoint being a change in MADRS total score from baseline (day one) to day 28. Treatment with ESK plus OAD was associated with a significantly greater change in MADRS score compared to placebo plus OAD	Safety assessment performed via physical examination, nasal examination, cognitive testing, CSSRS, CADSS, BPRS+, MOAA/S, PWC-20, Global Assessment of Discharge Readiness. Most common side effects were dissociation, dizziness, vertigo, dysgeusia, and they were more frequently observed in ESK plus OAD group
Ochs-Ross et al. (2020) [19] TRANSFORM-3	Randomized, multi-center, double-blind, and active-controlled; flexible dosing; arms = ESK (28 mg or 56 mg or 84 mg) plus OAD vs placebo plus OAD; N = 138 (ESK plus OAD = 72; placebo plus OAD = 66)	Adults with TRD; age group ≥ 65 years; treatment phase of four weeks, follow up for 24 weeks or entry into SUSTAIN-2	ESK 28 mg or 56 mg or 84 mg given intranasally two times per week	MADRS was used to assess efficacy, primary efficacy endpoint being a change in MADRS total score from baseline (day one) to day 28. No statistically significant difference was seen between treatment with ESK plus OAD group compared to placebo plus OAD group	Safety assessment performed via physical examination, nasal examination, cognitive testing, CSSRS, CADSS, BPRS+, MOAA/S, PWC-20, Global Assessment of Discharge Readiness. Dizziness, nausea, transient elevation in BP, fatigue, headache, dissociation were the common TEAEs. Safety profile was comparable to other similar studies done in younger adults
Daly et al. (2019) [20] SUSTAIN-1	Randomized withdrawal design, double-blind, multi-center, active-controlled; arms = fixed or flexible dose ESK (56 mg or 84 mg) plus OAD vs placebo plus OAD; N = 705 (direct entry = 437; transferred entry = 268); patients with stable remission during maintenance phase: N = 176 (ESK plus OAD = 90; placebo plus OAD = 86); patients with stable response without remission during maintenance phase: N = 121 (ESK plus OAD = 62; placebo plus OAD = 59)	Adults with TRD; age group = 18 to 64; direct-entry patients: four weeks of induction phase with flexible dosing (56 mg or 84 mg intranasally twice a week), followed by 12 weeks of optimization phase (in those achieving treatment response) with dosing same as the induction phase given once per week for four weeks and then once every two weeks or weekly. Transferred-entry responders (from TRANSFORM-1 and TRANSFORM-2): 12 weeks of optimization with the frequency of dosing, same as that mentioned for direct-entry patients. Maintenance phase: patients showing stable remission*** and patients showing stable response without remission**** randomized (1:1) to either continue ESK plus OAD or switch to placebo plus OAD		MADRS used, and the relapse time was assessed between the two treatment arms. Significantly delayed relapse of depressive symptoms observed in ESK plus OAD group	Safety assessment performed via physical examination, nasal examination, cognitive testing, CSSRS, CADSS, BPRS+, PWC-20. Side effects such as dissociation, vertigo, dizziness, dysgeusia. and somnolence were reported more frequently in ESK plus OAD group
Wajs et al. (2020) [21] SUSTAIN-2	Long-term (one year) study, multi-center, uncontrolled; flexible dosing; N=802 (direct entry = 691; transferred entry from TRANSFORM-3 = 111)	Adults with TRD; age group ≥18 years; direct entry patients: ESK 28 mg (for ≥ 65 years), 56 mg or 84 mg given intranasally twice weekly during the four-week induction phase (given along with OAD), and the responders continued with treatment once weekly or every other week for a 48-week optimization/maintenance phase, followed by a four-week follow-up. Transferred-entry responders: ESK 28mg or 56 mg or 84 mg (along with OAD) once weekly or every other week for 48-week optimization/maintenance phase followed by a four-week follow-up		MADRS scale used for efficacy evaluation. Improvement in depressive symptoms was found to be sustained in patients with TRD	Safety assessment performed via physical examination, nasal examination, cognitive testing, CSSRS, CADSS, BPRS+, PWC-20, BPIC-SS. Most TEAEs were of mild to moderate severity and included dizziness, dissociation, nausea, and headache

**Table 3:** Summary of post hoc studies, case/non-case study, and review NNT: number needed to treat; NNH: number needed to harm; LHH: likelihood of being helped or harmed; ESK: esketamine; OAD: oral antidepressant; FDA: Food and Drug Administration; FAERS: FDA Adverse Event Reporting System; AE: adverse effects; MADRS: Montgomery-Asberg Depression Rating Scale

Author/year of publication	Study type	Study characteristics	Results		
			Efficacy		Safety
Citrome et al. (2020) [22]	Post hoc study	Four phase-three, double-blind studies were used to collect data (TRANSFORM 1, TRANSFORM 2, TRANSFORM 3, SUSTAIN 1); NNT and NNH were calculated for efficacy outcomes and tolerability outcomes, respectively for ESK plus OAD vs placebo plus OAD in each study. LHH calculated; pooled results calculated for acute studies	NNT for efficacy outcomes for ESK plus OAD vs placebo plus OAD were less than 10	AEs with NNH value of less than 10 were dissociation, nausea, dizziness, vertigo, and dysgeusia. Use of ESK plus OAD was three times more likely to result in acute remission as opposed to discontinuation due to side effects	
Ochs-Ross et al. (2021) [23]	Post hoc study	This was a post hoc descriptive analysis used to compare the safety and tolerability of ESK in two treatment groups: TRD patients aged 18-64 vs TRD patients aged ≥ 65 in SUSTAIN-2 study	Treatment outcomes of ESK in both the age groups were comparable in terms of change in MADRS scores as well as response/remission rates	Treatment-emergent acute hypertension (TEAH) was observed more frequently in the elderly (age ≥ 65) patients. Except for this, the remaining findings on safety/tolerability of ESK were found to be comparable in both groups	
Gastaldon et al. (2019) [24]	Post hoc study	Four phase-three clinical trials were reviewed (TRANSFORM-1, TRANSFORM-2, TRANSFORM-3, SUSTAIN-1). Efficacy re-analysis, as well as re-analysis of the incidence of dissociation, was done on three short-term phase-three clinical trials (TRANSFORM-1, TRANSFORM-2, TRANSFORM-3)	Efficacy re-analysis showed a reduction of MADRS by 4 points. However, the clinical meaningfulness of this result remains unknown	Re-analysis showed that the occurrence of dissociation was seven times higher in the ESK group compared to placebo. 25% of patients receiving ESK experienced severe dissociation during treatment	
Gastaldon et al. (2021) [13]	Case/non-case study	FAERS database (March 2019-March 2020) containing 2274 esketamine-related side effects in 962 patients was used to evaluate safety signals of esketamine. In this case/non-case study design, cases included the AE reports where ESK was recorded, whereas non-cases included the AE reports of all the other drugs recorded in FAERS. Disproportionality was then tested to see if the AE were more commonly present in cases vs non-cases	Signals were detected for several side effects including dissociation, feeling drunk, sedation, depression, euphoric mood, suicidal ideation, and completed suicide. The study detected some rare AE which include self-harm ideation, increased loquacity, panic attacks, ataxia, mania, akathisia, and autoscopy.		
Horowitz and and Moncrieff (2020) [25]	Narrative review	The study reviewed the efficacy and safety of ESK based on the results of trials submitted to regulators including the FDA	In addition to uncertainties on long-term safety, the evidence regarding efficacy of ESK also remains scarce		

Discussion

Pharmacological Basis

**Figure 2:**
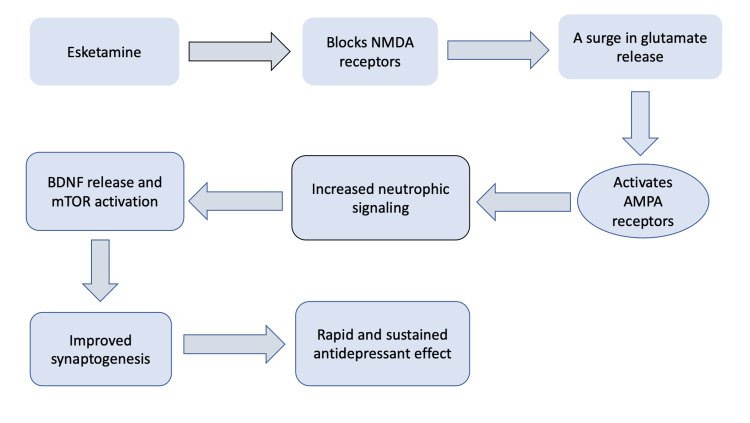
Proposed mechanism of action of esketamine NMDA: N-methyl-D-aspartate; AMPA: α-amino-3-hydroxy-5-methyl-4-isoxazolepropionic acid; BDNF: brain-derived neurotrophic factor; mTOR: mammalian target of rapamycin

The mechanism of action of ketamine as an anesthetic has been well researched. However, much remains unknown about the basis of antidepressant effects of esketamine. One of the proposed mechanisms includes improvement in brain plasticity (via increased neuronal dendritic growth and improved synaptogenesis) by stimulating the production of brain-derived neurotrophic factor (BDNF) and by activating the mammalian target of rapamycin (mTOR) [26-28]. Studies show that ketamine has a more direct stimulating action on BDNF and mTOR compared to oral antidepressants [27,29]. The same may apply to esketamine, and could explain the reason for its rapid onset of action, and the continuation of its effects even after elimination of the drug from the body [27,30]. The intranasal form of esketamine has multiple positive benefits as opposed to other modes of administration, as it is less painful and invasive, while also having a greater bioavailability than oral form [30]. Figure 2 shows the proposed mechanism of action of esketamine.

Efficacy of Esketamine

Out of the three short-term (four weeks) induction studies (TRANSFORM-1 [17], TRANSFORM-2 [18], TRANSFORM-3 [19]) included in this review, only TRANSFORM-2 showed a statistically significant reduction in Montgomery-Asberg Depression Rating Scale (MADRS) in esketamine plus OAD group compared to placebo plus OAD group, with least squared mean difference (LSMD) of −4.0 (standard errror (SE) = 1.69, 95% confidence interval (CI) = −7.31 to −0.64, p = 0.020). The mean difference in MADRS scoring between esketamine plus OAD vs placebo plus OAD in TRANSFORM 1 (LSMD = −3.2, 95% CI = −6.88 to 0.45; p = 0.088) AND TRANSFORM 3 (LMSD = −3.6, 95% CI = −7.20 to 0.07, p = 0.059) was similar to TRANSFORM-2 but failed to yield a statistical significance.

Clinically significant treatment effect was reported to be present in all three short-term studies [17-19]. These studies have taken a mean difference of 2 points or higher in the MADRS score between two treatment arms (esketamine plus OAD vs placebo plus OAD) as the acceptable cut-off to define clinically meaningful benefit [17-19]. However, there seem to be variations when it comes to defining “clinically meaningful improvement” among other experts, and this is one of the reasons why clinical relevance of these results has been debated [24-25]. On a post hoc analysis done by Gastaldon et al. [24], re-analysis was performed on the above mentioned clinical trials which showed a mean difference in MADRS score between esketamine plus OAD vs placebo plus OAD to be −4.08 (95% CI = −6.20 to 1.97), but the clinical meaningfulness of the result was stated to be uncertain. All three studies were similar in terms of study design including inclusion/exclusion criteria, but differed in dose regimen and age criteria (TRANSFORM-1 and TRANSFORM-2: age 18-64; TRANFORM-3: age ≥ 65 years) (Table 2).

SUSTAIN-1 [20] was a relapse prevention study based on withdrawal design where patients who achieved stable remission or stable response were randomized to either continue esketamine or discontinue it and switch to placebo nasal spray, and subsequent relapse was measured between the two groups. Among the stable remitters, risk of relapse decreased by 51% in those receiving esketamine plus OAD (hazard ratio [HR] = 0.49, 95% CI = 0.29-0.84, p = 0.003) compared to placebo plus OAD. Among the stable responders, risk of relapse decreased by 70% in those receiving esketamine plus OAD (HR = 0.30, 95% CI = 0.16-0.55, p<0.001) compared to placebo plus OAD. However, this study has received several feedbacks from other researchers, with one of their biggest concerns being the study design itself [24-25]. Some experts argue that the effects experienced as a result of withdrawal from esketamine can be mistaken for relapse of depressive symptoms, thereby confounding the result on relapse rate [24-25].

SUSTAIN-2 [21] was a phase-three long-term (up to a year) study used to assess long-term efficacy and safety of esketamine and included patients of age group ≥18 years. The percentage of remitters and responders at the end of induction phase was 47.2% and 78.4%, respectively. Similarly, at the end of optimization/maintenance phase was 58.2% and 76.5%, respectively. In terms of efficacy, sustained improvement in depressive symptoms was reported among responders and in those who continued the treatment for up to a year. A post hoc study was conducted by Ochs-Ross et al. [23] which compared the safety and tolerability of esketamine between young TRD patients aged 18-64 and older TRD patients aged ≥65 years included in SUSTAIN-2. Following the induction phase, mean change in the MADRS score was reported to be −16.6 in the younger age group and −15.4 in the older age group, with the remission rate being 46.5% and 50.0%, respectively. Similarly, the remission rate at the end of optimization/maintenance phase was reported to be 56.6% and 64.3% in the younger and older age groups, respectively. Thus, the efficacy of esketamine between the two age groups was found to be comparable.

Moreover, a post hoc study conducted by Citrome et al. [22] using data from four randomized clinical trials [17-20] showed that Number Needed to Treat ( NNT) for efficacy outcome for esketamine vs placebo was less than 10, which indicates esketamine to be a potentially effective treatment for TRD.

Safety of Esketamine

The most commonly reported adverse effects (AE) in short-term clinical trials [17-19] were nausea, dizziness, dissociation, headache, vertigo, and dysgeusia. Most of the AE were mild to moderate in severity and resolved the same day following dosing. In all three studies, the dissociative symptoms were seen shortly after dosing, which peaked at 40 minutes, and resolved in 1.5 hours. In a re-analysis done by Gastaldon et al. [24], the occurrence of dissociation was found to be seven times higher in esketamine plus OAD group compared to placebo plus OAD group, and around 25% of patients receiving esketamine were reported to have experienced dissociation during treatment. No symptom of psychosis was reported.

All three short-term studies [17-19] showed a greater mean increase in systolic as well as diastolic blood pressure (BP) in esketamine plus OAD group compared to placebo plus OAD group. For instance, in TRANSFORM-2, the mean maximum increase in systolic BP was +11.6 mmHg and +5 mmHg in esketamine plus OAD group and placebo plus OAD group, respectively and mean increase in diastolic BP was +8.1 mmHg and +4.5 mmHg in the two treatment groups, respectively. Similarly, a greater percentage of patients from esketamine plus OAD group reported moderate to greater sedation when compared to placebo plus OAD group in all three studies. During two weeks of follow-up, no withdrawal symptoms were observed after the discontinuation of esketamine plus OAD. The safety concerns of esketamine as reported by the FDA, which require Risk Evaluation and Mitigation Strategies (REMS) not only include dissociation and sedation but also misuse and abuse. Although no misuse or abuse was seen in any of the short-term studies, it is important to note that these studies were conducted in highly specialized centers with strict supervision [24]. Therefore, the possibility of such occurrences (misuse or abuse) in real-world setting shouldn’t be dismissed.

The most common symptoms reported in relapse prevention study and long-term clinical study [20-21] were dysgeusia, dissociation, vertigo, dizziness, and somnolence. Similar to the results of short-term clinical trials, these symptoms were mild to moderate in severity and most resolved on the same day of dosing. No respiratory depression or interstitial cystitis were observed in these studies. During the induction phase in SUSTAIN-1, the serious side effects considered to be due to esketamine included dysautonomia, hypothermia, disorientation, lacunar stroke, simple partial seizure, and sedation [20]. No death was reported in this study. On the contrary, in SUSTAIN-2, two deaths were reported. One of the deaths was due to respiratory and cardiac failure and the other death was due to suicide. Psychotic-like symptoms following dosing were found to be transient and resolved the same day. Dissociative symptoms pattern in both the studies was comparable to short-term studies, and were observed shortly after dosing, peaked at 40 minutes, and resolved by 1.5 hours. In SUSTAIN-1, no withdrawal symptoms were observed, whereas in SUSTAIN-2, the most common withdrawal symptoms following discontinuation of esketamine at the endpoint were insomnia (22.7%), anxiety/nervousness (19.3%), difficulty concentrating/remembering (19.3%), and dysphoric mood-depression (18.2%) [21].

A post hoc study by Citrome et al. [22] reported that the use of esketamine plus OAD was three times more likely to result in acute remission rather than discontinuation as a result of side effects. Similarly, it was reported that the side effects with Number Needed to Harm (NNH) values of less than 10 included dissociation, nausea, vertigo, dizziness, and dysgeusia. Thus, these AEs were reported to be more common and can be expected to occur as frequently as the treatment response itself. Likewise, in a post hoc study [23], safety/tolerability profile of esketamine was found to be comparable between younger age group (18-64) and older age group (≥65 years) except for the treatment-emergent AE of acute hypertension which was observed more frequently in the older age group.

In addition to the above mentioned AE, a case/non-case study [13] detected some rare AEs, which have not been reported by most studies. These include panic attacks, ataxia, mania, akathisia, self-harm ideation, autoscopy, and increased loquacity. Signals were detected for several side effects including dissociation, sedation, feeling drunk, euphoric mood, depression, suicidal ideation, and completed suicide. This study also reported that most of the serious side effects were found to be dose-dependent, and were more likely to occur in females and those receiving multiple antidepressants, benzodiazepines, antipsychotics, mood stabilizers, and somatic treatments.

Due to concerns regarding some of the AEs of esketamine, after licensing of esketamine the FDA has recommended the REMS, which requires the drug to be given in a specialized healthcare setting under strict monitoring for two hours after drug administration [25]. However, several researchers have placed some concerns regarding certain safety signals, which they felt were not sufficiently addressed by the FDA. In a review done by Horowitz and Moncrieff [25], several of the concerns regarding the clinical trials submitted to the FDA have been highlighted. For instance, there was one death due to motor vehicle accident in TRANSFORM-2 in a patient receiving esketamine, which was reported to be unrelated to esketamine. However, the reviewer argues that impaired hand-eye coordination and dissociation can increase the risk of road traffic accidents in ketamine users. Similarly, two deaths were reported in SUSTAIN-2, one was due to acute respiratory and cardiac failure and the other death was due to suicide; both were stated not to be due to esketamine. Again, based on previous studies of ketamine, increased BP has been shown to result in heart failure as well as myocardial infarction in those who are at risk [31-34]. Moreover, the death due to suicide, as reported in SUSTAIN-2, occurred in a patient with no previous history of suicidal behavior or intent, and the patient was also in clinical remission during this occurrence. Based on the review, there’s a possibility that these adverse outcomes could be linked to esketamine, and therefore requires careful attention and further studies [25].

Limitations

Most of the clinical trials mentioned in this review excluded patients with several significant medical/psychiatric comorbidities, those with a history of substance use disorder, and MDD patients who are at imminent risk of suicide. There was also a limited number of non-White patient inclusion. This has led to limitation in the generalizability of the results. Similarly, AEs of esketamine such as dissociation and sedation can lead to potential unblinding, in many of the clinical trials, which is important to be noted. Furthermore, three of the studies included in this review are post hoc studies which can have inherent bias. Additionally, since our study excluded gray literature, ongoing clinical trials on esketamine were not included, which could have been potentially useful in reaching out additional conclusions.

## Conclusions

Esketamine appears to be effective in reducing depressive symptoms in TRD patients and has a decent safety profile based on the results of the clinical trials. However, the clinical relevance of the treatment effect and the safety demonstrated by most clinical trials cannot be guaranteed in the real-world setting. First, there’s a paucity of long-term clinical trials on esketamine due to which its efficacy and safety on a long-term basis is still uncertain. Similarly, the superiority in the efficacy of esketamine over the pre-existing treatment modalities for TRD is also questionable due to the lack of comparative clinical trials so far. Although, most clinical trials have reported transient mild to moderate AEs of esketamine, new data are emerging which suggest the likelihood of its association with rare but potentially serious side effects. Therefore, in addition to strict post-marketing monitoring of esketamine, more robust and long-term randomized controlled clinical trials are needed to get a better insight into its safety and efficacy.
